# The effectiveness of waxing or epilation compared to conventional methods of hair removal in reducing the incidence of surgical site infections: a systematic review and meta-analysis

**DOI:** 10.3389/fsurg.2024.1395681

**Published:** 2024-12-06

**Authors:** Joseph Cutteridge, Pierre Garrido, Tim Staniland, Arthur Lim, Joshua Totty, Ross Lathan, George Smith, Ian Chetter

**Affiliations:** ^1^ Department of Health Sciences, Faculty of Sciences, University of York, York, United Kingdom; ^2^ Academic Vascular Surgical Unit, Hull University Teaching Hospitals NHS Trust, Hull, United Kingdom; ^3^ Department of General Surgery, Surrey and Sussex Healthcare NHS Trust, Redhill, United Kingdom; ^4^ Library & Knowledge Services, Hull University Teaching Hospitals NHS Trust, Hull, United Kingdom; ^5^ Centre for Clinical Sciences, Hull York Medical School, Hull, United Kingdom

**Keywords:** surgical site infection, epilation, waxing, hair removal, systematic review

## Abstract

**Background:**

Surgical site infections (SSIs) pose a significant challenge to healthcare systems by elevating patient morbidity and mortality and driving up financial costs. Preoperative skin preparation is crucial for preventing SSIs; however, certain traditional methods of hair removal have been found to increase the risk of SSI development. Mechanical epilation and waxing constitute two relatively explored methods of hair removal, which may hold potential to accelerate wound healing due to the activation of stem cells within hair follicles. This review assesses the efficacy of preoperative hair removal via waxing and mechanical epilation in reducing SSI incidence.

**Methods:**

This systematic review was prospectively registered with PROSPERO (ref: CRD42023423798) and a protocol previously published in a peer-reviewed journal. All findings are reported according to PRISMA guidelines. A comprehensive search of Medline, Embase, CENTRAL, ClinicalTrials.gov and CINAHL. Inclusion criteria encompassed adult patients undergoing any surgical procedure, comparing waxing or epilation against other hair removal methods or no hair removal, with SSI incidence as the primary outcome. There was no restriction on study size or quality to ensure a comprehensive literature evaluation.

**Results:**

The review found no studies meeting the selection criteria out of 576 records screened.

**Discussion/conclusion:**

This review has identified no literature regarding the use of waxing and mechanical epilation as methods of preoperative hair removal. The lack of experimental evidence combined with the potential physiological advantages of these techniques indicate that this could be a valuable area of future research. These techniques may represent novel approaches to SSI prevention, particularly beneficial in high-risk surgical disciplines like vascular surgery.

**Systematic Review Registration:**

https://www.crd.york.ac.uk/prospero/display_record.php?RecordID=423798, PROSPERO (CRD42023423798).

## 1 Introduction

Surgical site infections (SSIs) represent a significant challenge to UK healthcare systems, accounting for up to one in seven hospital-acquired infections and contributing substantially to morbidity and mortality (1). Specifically, SSIs can increase mortality rates by 2–11 times (2), and in vascular surgery, patients who contract an SSI following lower limb revascularisation are twice as likely to require an amputation by six months (3). Beyond the immediate health impacts, SSIs lead to a cascade of broader adverse outcomes including prolonged hospital stays, protracted courses of antibiotics, heightened psychological distress, and significantly greater healthcare costs (4, 5). The additional attributable cost of a single SSI has been estimated at around £5,239 (6).

Preoperative hair removal was previously common surgical practice across the globe, including in vascular surgery (7, 8). However, evidence began to emerge in the 1980s that shaving may increase the risk of SSI development (9, 10). The most recent Cochrane review (2021) states that while hair removal using clippers or depilatory cream does not appear to increase the risk of SSIs, shaving with a razor does indeed raise infection risk. Current NICE guidelines specify that hair should be removed before surgery only if it obstructs the operation, not with the intention of preventing SSI (11). If necessary, hair removal should be performed on the day of the operation using electric clippers equipped with a disposable head (11).

Epilation involves the removal of hair at the root. It is commonly conducted by waxing or the use of a mechanical epilator. Neither are commonplace before surgical procedures; however, such techniques offer a potential physiological advantage due to the activation of stem cells within hair follicles (HF). HFs are known to house a large number of stem cells (12, 13), with recent research suggesting they substantially contribute to the neoepidermis in wounded skin, and that these HF-derived cells are particularly important for acute wound healing (14–16). In addition, there is a strong link between the hair cycle phase and wound healing—when HFs are in a growth phase, healing is accelerated (17). What is not known is whether the physiological processes associated with epilation can reduce SSI incidence. Epilatory techniques may also offer practical advantages, such as an extended hair-free period, which facilitates the ease of wound cleaning and the application of adhesive dressings.

However, epilatory techniques also have potential disadvantages. Both waxing and mechanical epilation are associated with intervention-related pain. Waxing also has been associated with several adverse complications, including burns, allergic contact dermatitis and even infection (18–21). Furthermore, the increased microtrauma seen with waxing and mechanical epilation could predispose to SSI formation; however, there is no current guidance to inform practice regarding epilation prior to surgery.

### 1.1 Objective

The purpose of this systematic literature review was to evaluate the current evidence for preoperative removal of hair using waxing and epilation to reduce surgical site infection.

## 2 Methods

This systematic review was conducted using the Joanna Briggs Institute Evidence Synthesis Checklist (22) and the Cochrane Handbook for Systematic Reviews of Interventions (23). Findings are reported according to the extension for Preferred Reporting Items for Systematic Reviews and Meta Analyses (PRISMA) guidelines (24). It has been conducted according to our pre-published protocol (25) which was prospectively registered with PROSPERO (ref: CRD42023423798).

### 2.1 Selection criteria

The selection criteria of this review is displayed in the PICOS format in Table 1.

**Table 1:** Selection criteria.

	Inclusion criteria	Exclusion criteria
Population	Adult population undergoing any surgical procedure (elective or emergency)	Paediatric population (age <18 years)
Intervention	Use of waxing or epilation for preoperative hair removal	–
Comparator	Any other method of preoperative hair removal, or no hair removal	–
Outcome	Incidence of SSI at 30 days according to any diagnostic criteria	–
Study design	Randomised control trials Quasi-randomised control trial Observational studies	Case reports, case series Systematic and narrative reviews Letters Abstract only articles

Limitations in sample sizes or quality of study were not applied, to ensure a comprehensive assessment of the literature. Systematic and narrative review articles were excluded, although reference lists of these articles were hand-searched.

### 2.2 Search methods for identification of studies

The search strategy was designed in conjunction with an information specialist (TS). It consisted of a comprehensive search of Medline, Embase, CENTRAL, ClinicalTrials.gov and CINAHL. An example search strategy for Medline can be seen below (Table 2). Additional articles were sought by handsearching the references of any included articles and excluded review articles. The full search strategy can be found within the Supplementary Material.

**Table 2:** Example medline search strategys.

exp hair removal/	AND	exp preoperative care/	AND	exp Surgical Wound Infection/
wax*.ab,ti.		exp preoperative period/		exp Surgical Wound Dehiscence/
shav*.ab,ti.		preoperative.ab,ti.		surgical infection.ab,ti.
epilat*.ab,ti.				surgical site infection.ab,ti.
exp epilation/				SSI.ab,ti.
depilat*.ab,ti.				exp postoperative complication/
exp depilatory agent/				exp wound infection/
				wound infection.ab,ti.

### 2.3 Selection of studies

**Figure 1:**
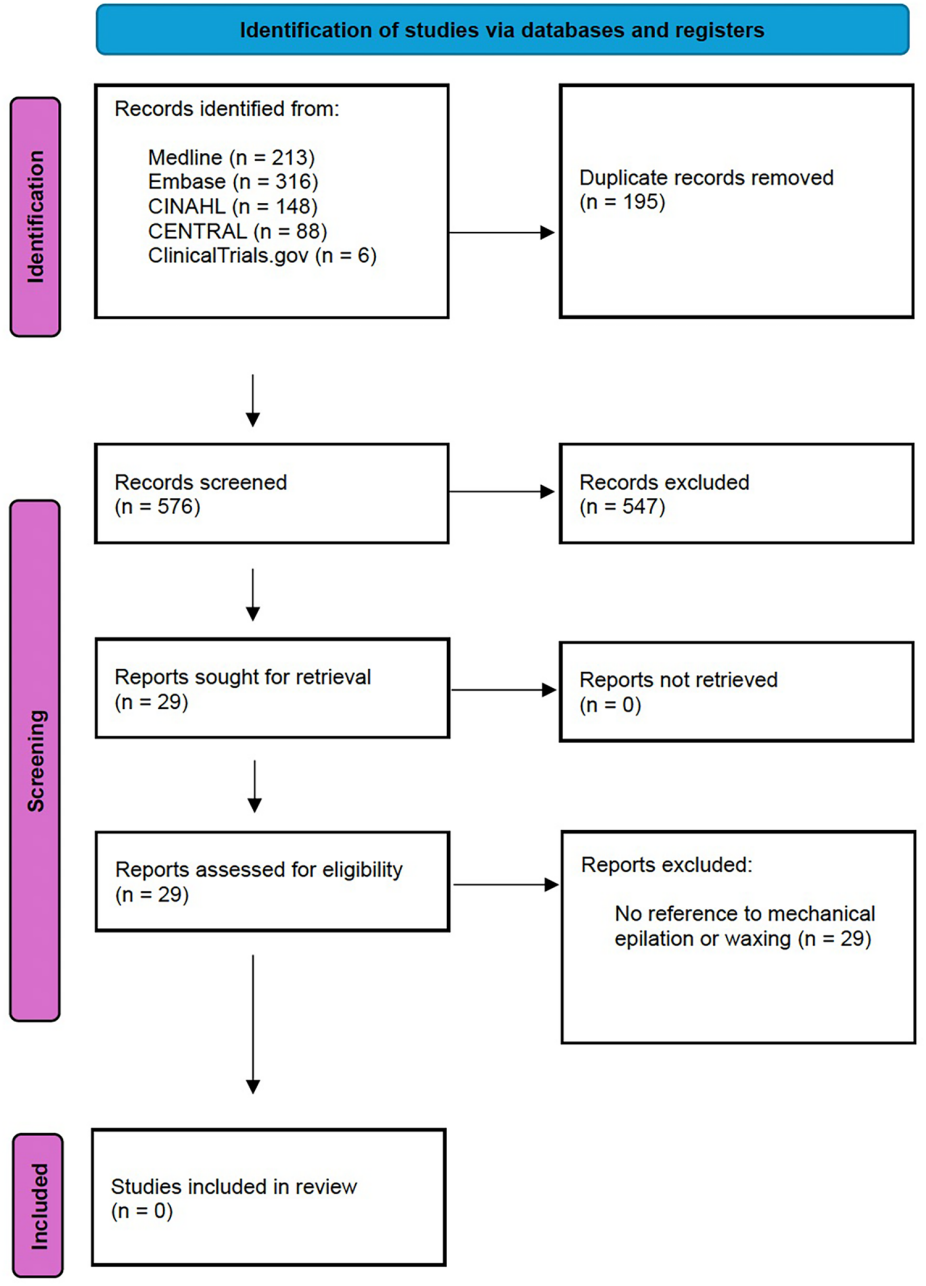
PRISMA flow chart.

Search results were uploaded to the Covidence online systematic review software, which automatically removed duplicate articles. Titles and abstracts were reviewed by two independent reviewers (JC and PG), screening against the selection criteria. English titles and abstracts were sourced for all foreign language papers. All conflicts were settled by a third author (RL) when required. All articles identified as relevant underwent assessment of the full-length manuscript. As previously, this was performed by two independent reviewers (JC and PG), with any disputes managed by a third (RL). The number of search hits, duplicates removed, full texts reviewed, articles excluded (with reasons), and the final number of studies included can be seen in Figure 1.

## 3 Results

No studies met our inclusion criteria (Figure 1). Of the 576 records screened, 29 articles were selected to undergo full-length text review. No foreign language papers were eligible for full-length text review based on their English titles and abstracts. All full-length texts of included articles were obtained and reviewed. However, none referred to mechanical epilation or waxing. A single article referenced epilatory hair removal, (Talero et al. 2019) (26), but within a very specific context of plucking of infected eyelashes. This method of hair removal did not form part of our inclusion criteria. Furthermore, the article did not report the incidence of surgical site infection as an outcome. Therefore, the article was excluded from analysis.

No additional articles were sourced from references of excluded review articles.

## 4 Discussion

This comprehensive, systematic literature search has shown that there is no interventional or observational evidence evaluating the preoperative removal of hair using waxing or epilation to reduce surgical site infection.

Many articles evaluated depilatory creams. However, this method of hair removal does not result in hair being removed by the root, i.e., it is not true epilation like mechanical epilation or waxing. Depilatory creams use alkaline-based compounds that break down keratin within hair. They result in the hair shaft breaking just below the skin's surface, enabling easy removal by wiping. Because the hair follicle is undisturbed, there is no reason to suspect that we would see the same level of stem cell activation as compared to epilatory methods, which put the HFs into a growth phase and accelerate healing (14–17). Therefore, we did not include depilatory creams as part of our inclusion criteria, with the majority of these records being excluded during title and abstract screening.

The article by Talero et al. refers to the plucking of eyelashes infected with Chlamydia Trochomatis, the cause of Trachomatous Trichiasis (TT), the most common infective cause of blindness worldwide (26). This specific form of epilation is recommended by the WHO in cases when patients cannot undergo TT surgery (26). The paper makes no reference to surgical site infections, so there is little relevant information that can be gleaned from this article. However, plucking does represent a method of epilation that was not considered in this review. This was primarily because the time needed to pluck all the hairs around the operative site would be very long, resulting in poor feasibility, so it seemed highly unlikely that any research would have been conducted in this area.

This study does have several limitations. Firstly, we must consider that because we have found no relevant studies, there is potential for publication bias. It's conceivable that studies in this field may exist but were not published due to non-significant or negative results. However, the addition of ClinicalTrials.gov within our search strategy helps mitigate this risk. Whilst we screened all foreign language papers returned from our search, we did not conduct alternatives searches in languages other than English. However, since most journals offer at least English titles to increase accessibility of their articles, we believe the likelihood of missing relevant articles is low. Finally, given that we have found no relevant articles, there are limitations in the conclusions we can draw from this study. Yet, this empty search does clearly highlight a significant gap in the literature.

Given that this review has failed to find any studies that meet our inclusion criteria, we can conclude that waxing and mechanical epilation constitute relatively unexplored areas of SSI prevention. However, to establish whether these hair removal methods hold the potential to reduce SSI rates, we require further research on a range of issues. One such avenue of research should involve gaining greater insight into the physiological process that occurs following epilation *in man*, as our current understanding is almost entirely derived from murine models (13, 14). Additionally, research to assess the safety and feasibility of these methods in patients is required, given several adverse reactions to waxing documented in the literature, including allergic contact dermatitis and burns (18, 19). Therefore, early clinical work must assess whether these interventions are indeed safe, and if they are feasible in the preoperative setting. For instance, excessive patient refusal due to discomfort or prolonged hair removal time in theatre would greatly limit the feasibility of these interventions. However, the widespread adoption of these products in the commercial sphere stands as a testament to their acceptability, at least among the general public (27, 28).

In summary, this review has identified no literature regarding the use of waxing and mechanical epilation as methods of preoperative hair removal. Despite widespread use in other contexts, these methods have not been properly investigated in the preoperative setting. Research is needed to determine if these hair removal techniques represent novel methods of SSI prevention.

## Data Availability

The original contributions presented in the study are included in the article/Supplementary Material, further inquiries can be directed to the corresponding author/s.
