# Supplementary material for: The effectiveness of waxing or epilation compared to conventional methods of hair removal in reducing the incidence of surgical site infections: a systematic review and meta-analysis
Source: Front Surg. 2024 Dec 6;11:1395681. doi: 10.3389/fsurg.2024.1395681 (PMC11659287; doi:10.3389/fsurg.2024.1395681)
Supplement: Supplementary file 1 [file Supplementaryfile1.docx]

Supplementary Material

The effectiveness of waxing or epilation compared to conventional methods of hair removal on reducing the incidence of surgical site infections: a systematic review and meta-analysis

Joseph Cutteridge^1,2*^, Pierre Garrido^3^, Tim Staniland^4^, Arthur Lim^2^, Joshua Totty^5^, Ross Lathan^2,5^, George Smith^2,5^, Ian Chetter^2,5^

1. Department of Health Sciences, Faculty of Sciences, University of York, York, UK
2. Academic Vascular Surgical Unit, Hull University Teaching Hospitals NHS Trust, Hull, UK
3. Surrey and Sussex Healthcare NHS Trust, Redhill, UK
4. Library & Knowledge Services, Hull University Teaching Hospitals NHS Trust, Hull, UK
5. Centre for Clinical Sciences, Hull York Medical School, Hull, UK

*** Correspondence:**[jdc578@york.ac.uk](mailto:jdc578@york.ac.uk)

## Supplementary Figures

| 1 | exp hair removal/ |
| --- | --- |
| 2 | wax*.ab,ti. |
| 3 | shav*.ab,ti. |
| 4 | epilat*.ab,ti. |
| 5 | exp epilation/ |
| 6 | depilat*.ab,ti. |
| 7 | exp depilatory agent/ |
| 8 | exp preoperative care/ |
| 9 | exp preoperative period/ |
| 10 | preoperative.ab,ti. |
| 11 | exp Surgical Wound Infection/ |
| 12 | exp Surgical Wound Dehiscence/ |
| 13 | surgical infection.ab,ti. |
| 14 | surgical site infection.ab,ti. |
| 15 | SSI.ab,ti. |
| 16 | exp postoperative complication/ |
| 17 | exp wound infection/ |
| 18 | wound infection.ab,ti. |
| 19 | 1 or 2 or 3 or 4 or 5 or 6 or 7 |
| 20 | 8 or 9 or 10 |
| 21 | 11 or 12 or 13 or 14 or 15 or 16 or 17 or 18 |
| 22 | 19 and 20 and 21 |

*Supplemental figure 1: Search strategy*

**
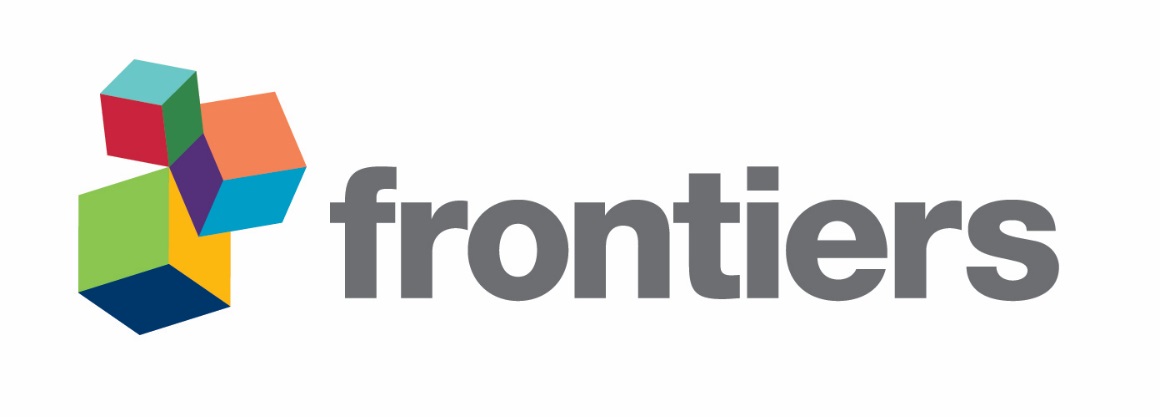
**
